# Medication Adherence in a Cross-Diagnostic Sample of Patients From the Affective-to-Psychotic Spectrum: Results From the PsyCourse Study

**DOI:** 10.3389/fpsyt.2021.713060

**Published:** 2022-01-20

**Authors:** Sophie-Kathrin Kirchner, Michael Lauseker, Kristina Adorjan, Heike Anderson-Schmidt, Ion-George Anghelescu, Bernhardt T. Baune, Monika Budde, Udo Dannlowski, Detlef E. Dietrich, Andreas J. Fallgatter, Peter Falkai, Christian Figge, Katrin Gade, Urs Heilbronner, Lena Hiendl, Georg Juckel, Janos L. Kalman, Farahnaz Klöhn-Saghatolislam, Carsten Konrad, Fabian U. Lang, Mojtaba Oraki Kohshour, Sergi Papiol, Daniela Reich-Erkelenz, Jens Reimer, Eva Z. Reininghaus, Sabrina K. Schaupp, Max Schmauß, Andrea Schmitt, Eva Christina Schulte, Simon Senner, Carsten Spitzer, Thomas Vogl, Jörg Zimmermann, Alkomiet Hasan, Thomas G. Schulze, Fanny Senner

**Affiliations:** ^1^Department of Psychiatry and Psychotherapy, University Hospital, Ludwig-Maximilians-University (LMU) Munich, Munich, Germany; ^2^Department of Psychiatry, Psychotherapy and Psychosomatics, Medical Faculty, Bezirkskrankenhaus (BKH) Augsburg, University of Augsburg, Augsburg, Germany; ^3^Institute for Medical Information Processing, Biometry, and Epidemiology, Ludwig-Maximilians-University (LMU) Munich, Munich, Germany; ^4^Institute of Psychiatric Phenomics and Genomics, University Hospital, Ludwig-Maximilians-University (LMU) Munich, Munich, Germany; ^5^Department of Psychiatry and Psychotherapy, University Medical Center Göttingen, Göttingen, Germany; ^6^Department of Psychiatry and Psychotherapy, Mental Health Institute Berlin, Berlin, Germany; ^7^Department of Psychiatry, University of Münster, Münster, Germany; ^8^Department of Psychiatry, Melbourne Medical School, The University of Melbourne, Melbourne, VIC, Australia; ^9^The Florey Institute of Neuroscience and Mental Health, The University of Melbourne, Parkville, VIC, Australia; ^10^AMEOS Clinical Center Hildesheim, Hildesheim, Germany; ^11^Center for Systems Neuroscience (ZSN), Hannover, Germany; ^12^Department of Psychiatry, Medical School of Hannover, Hannover, Germany; ^13^Department of Psychiatry and Psychotherapy, University Tübingen, Tübingen, Germany; ^14^Karl-Jaspers Clinic, European Medical School Oldenburg-Groningen, Oldenburg, Germany; ^15^Department of Psychiatry and Psychotherapeutic Medicine, Research Unit for Bipolar Affective Disorder, Medical University of Graz, Graz, Austria; ^16^Department of Psychiatry, Ruhr University Bochum, Landschaftsverbund Westfalen-Lippe (LWL) University Hospital, Bochum, Germany; ^17^International Max Planck Research School for Translational Psychiatry (IMPRS-TP), Munich, Germany; ^18^Department of Psychiatry and Psychotherapy, Agaplesion Diakonieklinikum, Rotenburg, Germany; ^19^Department of Psychiatry II, Ulm University, Bezirkskrankenhaus Günzburg, Günzburg, Germany; ^20^Department of Immunology, Faculty of Medicine, Ahvaz Jundishapur University of Medical Sciences, Ahvaz, Iran; ^21^Department of Psychiatry and Psychotherapy, University Medical Center Hamburg-Eppendorf, Hamburg, Germany; ^22^Department of Psychiatry, Health North Hospital Group, Bremen, Germany; ^23^Laboratory of Neuroscience (LIM27), Institute of Psychiatry, University of São Paulo, Butanta, Brazil; ^24^Department of Psychiatry and Psychotherapy, Technical University of Munich, School of Medicine, Munich, Germany; ^25^Department of Psychosomatic Medicine and Psychotherapy, University Medical Center Rostock, Rostock, Germany; ^26^Psychiatrieverbund Oldenburger Land gGmbH, Karl-Jaspers-Klinik, Bad Zwischenahn, Germany; ^27^Department of Psychiatry and Behavorial Sciences, SUNY Upstate Medical University, Syracuse, NY, United States

**Keywords:** medication adherence, schizophrenia, bipolar disorder, depression, conscientiousness

## Abstract

**Introduction:**

According to the World Health Organization, medication adherence is defined as the extent to which a person's behavior corresponds with an agreed recommendation from a healthcare provider. Approximately 50% of patients do not take their medication as prescribed, and non-adherence can contribute to the progress of a disease. For patients suffering from mental diseases non-adherence plays an important role. Various factors have been proposed as contributing to non-adherence, however the literature remains heterogeneous dependent on the analyzed patient subgroups. This study comprehensively evaluates the association of sociodemographic, clinical, personality and quality of life related factors with medication adherence by analyzing data from the PsyCourse study. The PsyCourse study is a large and cross-diagnostic cohort of psychiatric patients from the affective-to-psychotic spectrum.

**Methods:**

The study sample comprised 1,062 patients from the PsyCourse study with various psychiatric diagnoses (mean [SD] age, 42.82 [12.98] years; 47.4% female). Data were analyzed to identify specific factors associated with medication adherence, and adherence was measured by a self-rating questionnaire. Odds ratios (OR) were estimated by a logistic regression for binary outcomes. Missing data were imputed using multiple imputation.

**Results:**

The following factors showed the strongest association with medication adherence: never having used illicit drugs (OR, 0.71), number of prescribed antipsychotics (OR, 1.40), the personality trait conscientiousness (OR, 1.26), and the environmental domain of quality of life (OR, 1.09).

**Conclusion:**

In a large and cross-diagnostic sample, we could show that a higher level of conscientiousness, a higher number of antipsychotic medication, a better quality of life within the environmental domain, and the absence of substance abuse contribute to a better medication adherence independent of the underlying disorder.

## Introduction

Psychiatric disorders contribute 7% of the overall global burden of diseases, as measured in disability-adjusted life years, and 19% of all years lived with disability (1). The World Health Organization (WHO) has established a comprehensive mental health action plan to strengthen effective leadership and governance for mental health (2). Strengthening treatment adherence is one of the crucial aspects of the plan to secure sufficient treatment for mental health. Although useful drugs have been discovered for many psychiatric disorders, a substantial amount of patients do not take their medication regularly (3, 4). The consequences of non-adherence for the individual patient include relapses of symptoms, exacerbation of psychopathology, rehospitalization, prolonged disability, poor quality of life or psychosocial outcomes, and increased suicides (4). Additionally, non-adherence leads to increased co-morbid medical conditions and wastage of health care resources (3, 5).

According to a systematic review by Semahegn et al., 56% of patients with schizophrenia, 50% of patients with major depression, and 44% of patients with bipolar disorder are non-adherent (4). The WHO defines medication non-adherence as “a case in which a person's behavior in taking medication does not correspond with agreed recommendations from health personnel” (6). Non-adherence can have various reasons and can be intentional or unintentional. Especially in chronic psychiatric diseases such as bipolar disorder, unipolar depression, schizophrenia, and schizoaffective disorder, stabilization in the long-term often depends on good cooperation between the patient and health care provider and on treatment adherence (7, 8).

Reasons for non-adherence are usually multifactorial. Social support seems to play an important role, as patients from cohesive families and with practical support show higher treatment adherence (9). Disorder-related factors shape adherence behavior: a longer duration of illness favors non-adherence in both psychotic and affective disorders (10–13). Polypharmacy and adverse events were also predictors for lower adherence especially in patients suffering from bipolar and psychotic disorder (14–16). Patients with comorbid substance abuse show in various studies a lower adherence to their somatic and psychiatric medication (17–23). Research findings provide contradicting results on the association of general intelligence and adherence in patients with schizophrenia and bipolar disorder (24–27). Pronounced personality characteristics can complicate patients' interpersonal relationships, and perhaps cooperation in treatment (28). In patients suffering from early psychosis, high agreeableness is associated with poor medication adherence (29). In somatic disorders, studies showed that different personality traits mediate medication adherence, and in particular patients who score higher on conscientiousness are more adherent (30–32). Conscientiousness is defined as the propensity to follow socially prescribed norms for impulse control, to be goal directed, to plan, and to be able to delay gratification (33). In this respect, the question arises as to whether conscientiousness also influences treatment adherence in psychiatric disorders.

As summarized by Semahegn et al. (4), heterogeneous studies suggest that many factors influence treatment adherence in different disorders. By exploratively analyzing data from the PsyCourse study, this study aimed to investigate which sociodemographic, clinical, personality and quality of life-related factors are associated with treatment adherence in a group of 1,062 patients from the affective to psychotic spectrum.

## Methods

### Participants

Data were used from the longitudinal, multicenter PsyCourse study, which was conducted in Germany and Austria (www.PsyCourse.de) between 2011 and 2019. Diagnoses were assessed with parts of the structured clinical interview for DSM-IV. Participants were phenotyped by a comprehensive battery of tests that collected data on sociodemographics, illness history, treatment setting, psychopathology, cognition, functioning, personality traits, and quality of life. A detailed description of the study concept is available in the publication by Budde et al. (34). In our study, we used data from the first study visit, including all clinical participants with available information on adherence behavior; 161 participants had to be excluded because this information was missing. Healthy controls were not included. The sample comprised 1,062 participants with a DSM-IV diagnosis of schizophrenia, other psychotic disorder, schizoaffective disorder, bipolar disorder, or recurrent unipolar depression. This project analyzed data from the PsyCourse Phenotype Dataset, version 3.1. The study was approved by the local ethics committee and was performed in accordance with the Declaration of Helsinki.

### Adherence Instrument

Adherence was measured with a non-standardized self-rating questionnaire that assesses the regularity of medication intake similar to the Brief Adherence Rating Scale (BARS) (35). It asks whether the patient had taken their psychopharmacological medication as prescribed in the past seven days and past 6 months. Reponses for both items ranged from 1 to 6, as follows: 1, “every day, exactly as prescribed;” 2, ”every day, but not always as prescribed;” 3, “regularly, but not every day;” 4, ”sometimes, but not regularly;” 5, “seldom;” and 6, ”not at all.“ In the analyses, we used the responses about the past 6 months and grouped them into two superordinate categories: 1–2, ”daily intake of medication,“ and 3–6, ”irregular intake of medication.“ In the following, for better clarity we refer to patients with daily medication intake as *adherent patients* and to those with unregular medication intake as *non-adherent patients*.

### Sociodemographic Data

The mean [SD] age of participants was 42.82 [12.98] years; 47.4% of participants were female, and 52.6%, male. We collected information on family and partnerships, living situation, education, and work.

### Diagnoses, History of Illness, and Treatment Setting

The distribution of diagnoses was as follows: 39.4% schizophrenia, 8.2% schizoaffective disorder, 1.0% schizophreniform disorder, 0.6% brief psychotic disorder, 34.6% bipolar-I disorder, 9.1% bipolar-II disorder, and 7.3% unipolar depression. For the analyses, we grouped the diagnoses into three categories: psychotic disorder (schizophrenia, schizoaffective disorder, schizophreniform disorder, and brief psychotic disorder), bipolar disorder (bipolar I disorder, bipolar II disorder), and unipolar depression. The PsyCourse dataset also includes information on illness course, including the age of onset and duration; number of hospitalizations; current treatment setting; suicidal ideation and suicide attempts; and comorbidities such as addictive disorders. Functioning and severity of illness were measured with the Clinical Global Impression scale (CGI) (36) and the Global Assessment of Functioning (GAF) (37).

### Crystallized Intelligence Score

An approximate measure of general intelligence was obtained from participants in the PsyCourse study in the form of a crystallized intelligence assessed with the multiple-choice vocabulary intelligence test MWT-B (Mehrfachwahl-Wortschatz-Intelligenz Test) (38). In this test, participants are presented with 37 sets of five words each, four of which are “artificial words,” i.e., they do not exist in German. Participants are instructed to mark the real word, and the number of correctly identified real words is summed to give the final score.

### Personality Dimensions

To assess personality traits, the PsyCourse study used the Big Five Inventory-10 (BFI-10) questionnaire (39). The inventory is based on the well-known “Big Five” personality model, which comprises the five dimensions extraversion, neuroticism, conscientiousness, agreeableness, and openness. For the analyses, we calculated the sum scores for each personality dimension (40).

### Quality of Life

The WHOQOL-BREF was used to assess quality of life in the past 2 weeks (41). This instrument comprises 26 items that measure the following broad domains: physical health, psychological health, social relationships, and environment.

### Univariate Analysis

As mentioned above, medication adherence was assessed dichotomously (i.e., as adherent or non-adherent). Numerical and ordinal data were expressed as means [SD], and nominal data, as frequencies. Numerical and ordinal dependent variables were compared by the Mann-Whitney-U test for non-normally distributed measurements, and categorical data, by Chi-squared tests (Phi and Cramer's Test). An alpha value of 0.05 was considered significant. Statistical analyses were performed with IBM SPSS statistics, version 25.0.

### Multivariable Analysis

Missing values were multiply imputed using fully conditional specifications (MICE algorithm) (42), resulting in 10 (completed) data sets. Studies have been shown that more imputations do usually not lead to better results (43). The number of missing values is shown in Supplementary Table 1. A logistic regression model was used with the dichotomous outcome medication adherence and 45 variables available from the dataset as independent variables. Variable selection was performed with Akaike's information criterion in each of the completed data sets, resulting in 10 models. Variables were considered for the final model if they were selected in at least 5 of these 10 models. The model was re-run with these 14 variables, and regression coefficients were averaged with Rubin's rules. The analysis was performed with R 4.0.0, in particular the MICE packages (42).

## Results

### Univariate Analyses

Descriptive data of the study population is displayed in Table 1. One fifth (20.4%) of all patients reported non-adherent behavior within the last six months of treatment. The non-adherent patients were more often male (*p* = 0.016) and younger (*p* < 0.001). Descriptive analyses of sociodemographic, and clinical data in the adherent and non-adherent groups are summarized in Table 2.

**Table 1:** Descriptive data of study population.

	**Mean (SD) or *n* (%)**	**Min**	**Max**
**Sociodemographic data**			
Age	42.82 (12.98)	18	86
Male sex	559 (52.6)		
Relationship status: no partner	569 (55.1)		
Living alone	440 (41.4)		
High educational level: university entrance diploma	487 (46.2)		
Higher education: university degree	203 (19.3)		
Paid employment: not currently	631 (60.0)		
**Clinical data**			
Age at first outpatient treatment, *y*	28.46 (10.90)	4	73
Age at first inpatient treatment, y	30.2 (11.59)	5	73
Duration of illness, y	12.67 (10.70)	0	53
CGI	4.1 (1.04)	1	7
GAF	56.54 (13.44)	4	97
Current treatment: inpatient	430 (40.5)		
Diagnosis of alcohol dependency	111 (11.6)		
Patients with no or once monthly alcohol use	610 (57.44)		
Tobacco use: smoker	567 (53.6)		
Use of illicit drugs: yes	454 (44.6)		
Ever had suicidal ideation: yes	459 (77.1)		
Crystallized IQ: MWT-B	28.12 (4.98)	10	37
Total number of medication	2.44 (1.32)	0	8
Number of antidepressants	0.48 (0.64)	0	3
Number of antipsychotics	1.31 (0.97)	0	5
Number of mood stabilizers	0.42 (0.58)	0	3
Number of tranquilizers	0.21 (0.47)	0	2
Other psychotherapeutics	0.01 (0.12)	0	2
**Personality dimensions**			
Neuroticism	3.21 (0.97)	1	5
Extraversion	2.96 (1.06)	1	5
Openness	3.58 (1.02)	1	5
Conscientiousness	3.58 (0.87)	1	5
Agreeableness	3.46 (0.80)	1	5
**Quality of life**			
Global quality of life	12.56 (3.74)	4	20
Physical health	13.62 (2.78)	5.14	20
Psychological health	12.86 (3.32)	4.67	20
Social relationships	13.02 (3.50)	4	20

*Y, years; CGI, Clinical Global Impression; GAF, Global Assessment of Functioning, MWT-B, Mehrfachwahl-Wortschatz-Intelligenz Test*.

**Table 2:** Comparison of sociodemographic and clinical variables between the adherent and non-adherent patient groups.

	**Adherent patients** **(*n* = 845)**	**Non-adherent patients** **(*n* = 217)**	**Statistic**	***p*-value**	**Effect size**
	**Mean (SD) or *n* (%)**	**Mean (SD) or *n* (%)**			
**Sociodemographic data**					
Age, y	44.02 (11.78)	38.18 (12.24)	*U =* 67661.50, *z = –*5.96	**<0.001**	*r =* −0.18
Male sex	429 (50.7)	130 (59.9)	*X*^2^(1) = 5.78	**0.016**	*ϕ =* 0.073
Relationship status: no partner	439 (53.4)	130 (61.9)	*X*^2^(1) = 4.88	**0.027**	*ϕ =* 0.067
Living alone	343 (40.6)	97 (44.7)	*X*^2^(1) = 1.20	0.273	*ϕ =* 0.034
High educational level: university entrance diploma	392 (46.7)	95 (44.2)	*X*^2^(3) = 1.88	0.759	*ϕ =* 0.042
Higher education: university degree	172 (20.6)	31 (14.5)	*X*^2^(3) = 16.37	**0.003**	*ϕ =* 0.12
Paid employment: not currently	501 (59.9)	130 (60.5)	*X*^2^(1) = 0.03	0.876	*ϕ =* 0.005
Absence from work, months	12.23 (16.14)	13.07 (16.74)	*U =* 28891.50, *z = –*0.36	0.722	*r = –*0.01
**Clinical data**					
Age at first outpatient treatment, y	29.07 (10.99)	25.97 (10.13)	*U =* 62,782.50, *z = –*3.83	**<** **0.001**	*r = –*0.12
Age at first inpatient treatment, y	30.9 (11.78)	27.48 (10.42)	*U =* 67,985.50, *z = –*4.09	**<** **0.001**	*r = –*0.13
Duration of illness, y	13.23 (11.05)	10.48 (8.88)	*U =* 71,473.50, *z = –*2.91	**0.004**	*r = –*0.09
CGI	4.05 (1.05)	4.29 (0.99)	*U =* 78,426.00, *z = –*2.89	**0.004**	*r = –*0.09
GAF	57.26 (13.173)	53.77 (14.10)	*U =* 76,072.50, *z = –*3.41	**0.001**	*r = –*0.10
Current treatment: inpatient	301 (35.6)	129 (59.4)	*U =* 65,905.00, *z = –*6.66	**<** **0.001**	*r = –*0.20
Diagnosis of alcohol dependency	87 (11.6)	24 (11.7)	*X*^2^(1) = 0.001	0.974	*ϕ =* 0.000
Patients with no or once monthly alcohol use	494 (58.46)	116 (53.5)	*X*^2^(1) = 6.73	**0.035**	*ϕ =* 0.079
Tobacco use: smoker	429 (51.0)	138 (63.6)	*X*^2^(1) = 13.82	**0.001**	*ϕ =* 0.114
Use of illicit drugs: yes	335 (41.5)	119 (56.7)	*X*^2^(1) = 15.60	**<** **0.001**	*ϕ =* 0.121
Ever had suicidal ideation: yes	369 (76.7)	90 (78.9)	*X*^2^(1) = 0.26	0.610	*ϕ =* 0.016
Ever attempted suicide: yes	147 (30.6)	39 (33.9)	*X*^2^(1) = 4.01	0.135	*ϕ =* 0.061

*Bold values indicate significance (p < 0.05).*

*Y, years; CGI, Clinical Global Impression; GAF, Global Assessment of Functioning.*

*r, Pearson correlation coefficient: <0.3, small effect size; 0.3–0.5, medium effect size; > 0.5, large effect size.*

*ϕ, Phi coefficient: <0.25, small effect size; 0.25–0.66, medium effect size; >0.66, large effect size*.

The distribution of the diagnostic groups psychotic disorder and bipolar disorder was significantly different in the adherent and non-adherent groups (*X*^2^(1) = 12.67, *p* = 0.002, Φ = 0.109): Within the adherent group, 46.5% of the patients were diagnosed with a psychotic disorder, 46.3% with bipolar disorder, and 7.2% with unipolar depression. The respective percentages in the non-adherent group were 59.4, 33.2, and 7.4%.

Furthermore, the mean MWT-B score was higher in adherent patients than in non-adherent patients (adherent group, 28.31 [4.89]; non-adherent group, 27.29 [5.12]; *U* = 55,568.50; *z* = −2.34; *p* = 0.02).

The comparison of the number of medications prescribed and adverse events between adherent and non-adherent patient groups is summarized in Table 3.

**Table 3:** Comparison of the number of prescribed medications and adverse events in the adherent and non-adherent patient groups.

	**Adherent patients (*n* = 845)**	**Non-adherent patients (*n* = 217)**	**Statistic**	***p*-value**	**Effect size**
	**Mean (SD)**	**Mean (SD)**			
**Cross-diagnostic group**				
Antidepressants	0.51 (0.65)	0.43 (0.60)	*U =* 68267.50, *z = –*1.54	0.123	*r = –*0.047
Antipsychotics	1.38 (0.98)	1.25 (0.92)	*U =* 85064.50, *z = –*1.74	0.081	*r = –*0.053
Mood stabilizers	0.46 (0.61)	0.29 (0.48)	*U =* 78900.00, *z = –*3.73	**0.000**	*r = –*0.114
Tranquilizers	0.21 (0.46)	0.26 (0.50)	*U =* 86837.50, *z = –*1.76	0.079	*r = –*0.054
Other psychotherapeutics	0.02 (0.18)	0.01 (0.12)	*U =* 91538.00, *z = –*0.17	0.865	*r = –*0.005
Overall	2.58 (1.30)	2.24 (1.21)	*U =* 78411.50, *z = –*3.40	**0.001**	*r = –*0.104
**Psychotic disorder group**				
Antidepressants	0.34 (0.55)	0.29 (0.51)	*U =* 24386.00, *z = –*0.81	0.416	*r = –*0.025
Antipsychotics	1.84 (0.88)	1.56 (0.91)	*U =* 20854.50, *z = –*3.22	**0.001**	*r = –*0.099
Mood stabilizers	0.15 (0.40)	0.12 (0.32)	*U =* 24768.00, *z = –*0.67	0.506	*r = –*0.020
Tranquilizers	0.27 (0.50)	0.27 (0.50)	*U =* 25284.50, *z = –*0.06	0.954	*r = –*0.001
Other psychotherapeutics	0.01 (0.09)	0.02 (0.12)	*U =* 25149.00, *z = –*0.80	0.426	*r = –*0.025
Overall	2.62 (0.09)	2.26 (1.19)	*U =* 21324.00, *z = –*2.80	**0.005**	*r = –*0.056
**Bipolar disorder group**				
Antidepressants	0.57 (0.67)	0.53 (0.63)	*U =* 13711.50, *z = –*0.39	0.695	*r = –*0.012
Antipsychotics	1.03 (0.90)	0.92 (0.75)	*U =* 13390.50, *z = –*0.71	0.477	*r = –*0.022
Mood stabilizers	0.83 (0.60)	0.65 (0.56)	*U =* 12039.50, *z = –*2.27	**0.023**	*r = –*0.070
Tranquilizers	0.16 (0.44)	0.26 (0.53)	*U =* 12795.50, *z = –*2.01	**0.044**	*r = –*0.062
Other psychotherapeutics	0.03 (0.17)	0.01 (0.12)	*U =* 13947.00, *z = –*0.29	0.623	*r = –*0.009
Overall	2.62 (1.30)	2.38 (1.24)	*U =* 12725.00, *z = –*1.34	0.181	*r = –*0.041
**Unipolar depression group**				
Antidepressants	1.16 (0.64)	1.06 (0.68)	*U =* 454.50, *z = –*0.48	0.628	*r = –*0.015
Antipsychotics	0.66 (0.65)	0.25 (0.45)	*U =* 326.00, *z = –*2.28	**0.023**	*r = –*0.070
Mood stabilizers	0.10 (0.30)	0.06 (0.25)	*U =* 470.50, *z = –*0.44	0.659	*r = –*0.014
Tranquilizers	0.10 (0.30)	0.19 (0.00)	*U =* 444.50, *z = –*0.44	0.326	*r = –*0.014
Other psychotherapeutics	0.02 (0.13)	0	*U =* 480.00, *z = –*0.51	0.448	*r = –*0.016
Overall	2.03 (1.05)	1.56 (1.09)	*U =* 375.00, *z = –*1.47	0.458	*r = –*0.045
**Adverse events**				
Yes	404 (52.9)	85 (39.8)	*X*^2^(1) = 8.39	**0.004**	*Φ =* 0.089

*Bold values indicate significance (p < 0.05).*

*r, Pearson correlation coefficient: <0.3, small effect size; 0.3–0.5, medium effect size; >0.5, large effect size.*

*ϕ, Phi coefficient: <0.25, small effect size; 0.25–0.66, medium effect size; >0.66, large effect size*.

The analyses of the association of personality dimensions with adherence behavior were performed both across and within the diagnostic groups. In the cross-diagnostic sample, non-adherent patients scored significantly lower in the domain of conscientiousness (adherent group, 3.63 [0.85]; non-adherent group, 3.43 [0.91]; *U* = 66051.00; *z* = −2.74; *p* = 0.006). The separate analyses within each of the three diagnostic groups found no significant differences.

Adherent and non-adherent patients rated quality of life differently, i.e., non-adherent patients rated quality of life significantly lower in the domains *social relationships* (adherent group, 13.21 [3.34]; non-adherent group, 12.07 [3.97]; *U* = 63,397.00; *z* = –3.58; *p* < 0.001) and *environment* (adherent group, 15.05 [2.64]; non-adherent group, 13.97 [2.86]; *U* = 57,195.00; *z* = –4.68; *p* < 0.001). The association between the total quality of life score and medication adherence was marginally significant. Implying that non-adherent patients tended to experience a worse quality of life compared to adherent patients (adherent group, 12.66 [3.75]; non-adherent group, 12.15 [3.67]; *U* = 70,371.00; *z* = –1.96; *p* = 0.051). The quality of life in the domains *physical health* (adherent group, 13.62 [2.78]; non-adherent group, 13.39 [2.78]; *U* = 71,173.00; *z* = –0.83; *p* = 0.408) and *psychological health* (adherent group, 12.96 [3.31]; non-adherent group, 12.60 [3.24]; *U* = 68,890.50; *z* = –1.47; *p* = 0.143) was not significantly different between the two groups.

### Multivariable Analysis

**Figure 1:**
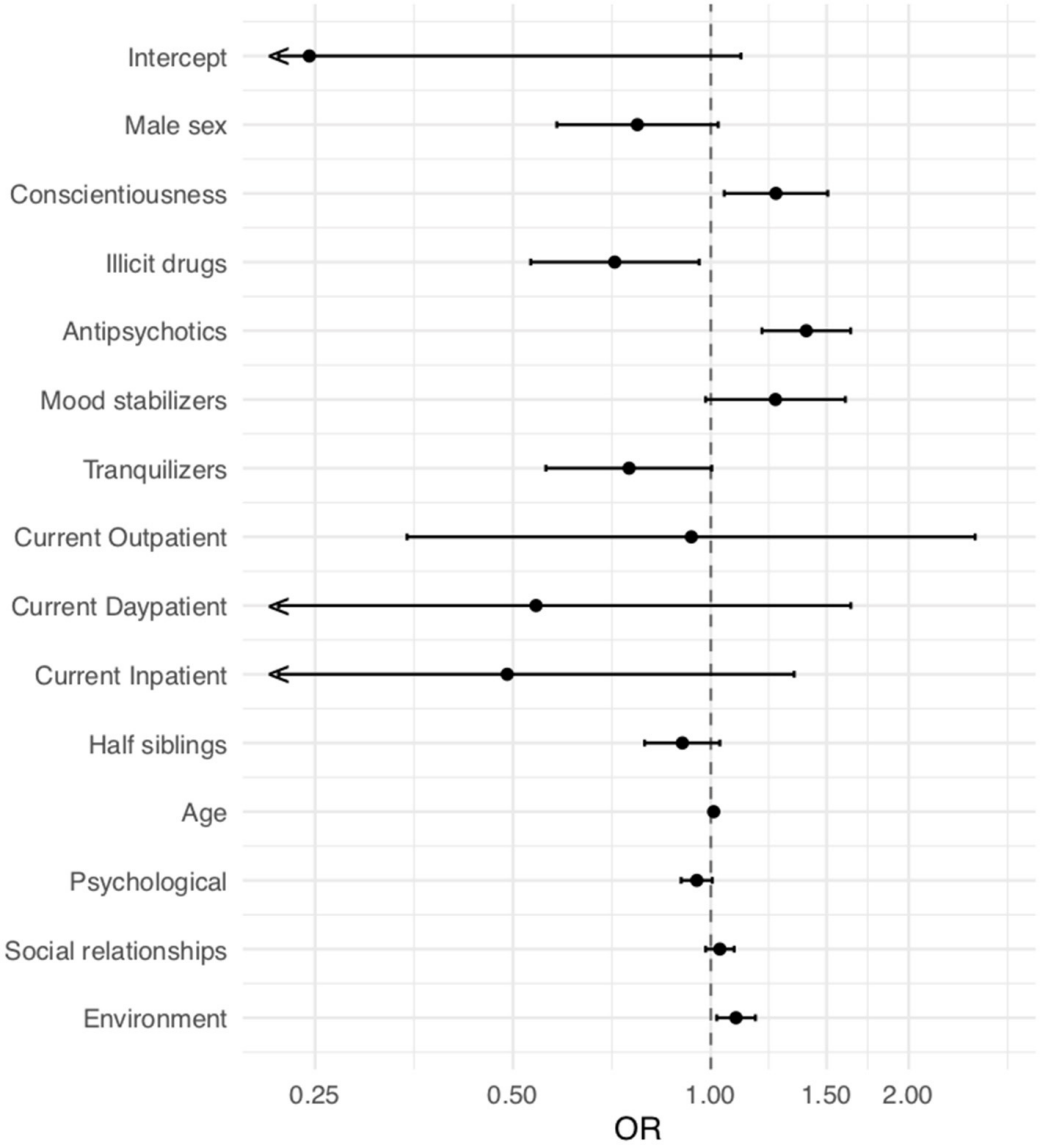
Odds ratios in the final model for adherence (*x* axis) and 95% CI. Intercept (odds ratio [OR], 0.26; 95% CI, 0.05–1.11; *p* = 0.069), male sex (OR, 0.77; 95% CI, 0.58–1.02; *p* = 0.075), conscientiousness (OR, 1.26; 95% CI, 1.05–1.51; *p* = 0.014), illicit drugs (OR, 0.71; 95% CI,: 0.53–0.96; *p* = 0.026), antipsychotics (OR, 1.40; 95% CI, 1.20–1.63; *p* < 0.001), mood stabilizers (OR, 1.25; 95% CI, 0.98–1.60; *p* = 0.069), tranquilizers (OR, 0.75; 95% CI, 0.56–1.00; *p* = 0.053), current outpatient status (OR, 0.93; 95% CI, 0.35–2.52; *p* = 0.893), current day-patient status (OR, 0.54; 95% CI, 0.18–1.63; *p* = 0.277), current inpatient status (OR, 0.49; 95% CI, 0.18–1.34; *p* = 0.165), half siblings (OR, 0.91; 95% CI, 0.79–1.03; *p* = 0.139), age [10 years] (OR, 1.11; 95% CI, 0.99–1.25; *p* = 0.077), quality of life—psychological (OR, 0.95; 95% CI, 0.90–1.01; *p* = 0.074), quality of life—social relationships (OR, 1.03; 95% CI, 0.98–1.09; *p* = 0.216), quality of life—environmental (OR, 1.09; 95% CI, 1.02–1.17; *p* = 0.011). O*R*, odds ratio.

To find factors potentially associated with medication adherence, we performed a logistic regression analysis. The odds ratios and confidence intervals of the final model are shown in Figure 1.

Independent of the main disorder, the personality trait conscientiousness (OR, 1.26; 95% CI, 1.05–1.51; *p* = 0.014) was significantly associated with medication adherence. The use of illicit drugs had a negative effect on medication adherence (OR, 0.71; 95% CI, 0.53–0.96; *p* = 0.026). The medication regimen seemed to play an important role in our sample, which consisted mainly of patients with psychotic and bipolar disorder. In particular, the number of prescribed antipsychotics was associated with medication adherence (OR, 1.40 per medication; 95% CI, 1.20–1.63; *p* < 0.001). Moreover, patients with a higher quality of life in the domain *environment* showed better medication adherence (OR, 1.09; 95% CI, 1.02–1.17; *p* = 0.011).

## Discussion

Medication adherence is a core component of adequate treatment of patients with psychiatric disorders. Previous studies found that medication adherence is affected mainly by demographic characteristics such as age and sex, social support system, comorbidities, substance abuse, and patients' individual attitude toward their medication and insight into their disorder (4). However, evidence investigating the association between personality traits and medication adherence in psychiatric patients remains sparse. In our large-scale, cross-diagnostic sample of chronically ill and well-phenotyped patients, we could identify conscientiousness, the absence of substance abuse and a higher number of antipsychotic medications, and a better quality of life in the domain environment as beneficial for medication adherence. These associations were found in the logistic regression analysis independent of the underlying disorder.

The positive association of conscientiousness and medication adherence is in line with other studies, where patients suffering from chronic somatic disorders or depression and with the prominent trait of conscientiousness were more adherent (44). Conscientious people tend to control impulses, delay gratification, set and reach goals, and plan in advance (45). Therefore, conscientious patients probably find it easier to manage the challenge of treating a chronic psychiatric disorder. Conversely, patients who score low on this personality trait might need more help to adjust to a regular medication regimen. Thus, considering personality traits may help to identify patients who need special support to adhere to their medication.

In line with other studies, we found that patients with a history of substance abuse have a higher risk of discontinuing their treatment regimen (20–23). Personality profiles with low conscientiousness are also associated with addictive disorders (46). Therefore, conscientiousness could be the mediator of this finding.

In our study, the adherent group was treated with a higher number of antipsychotic and mood-stabilizing drugs. This finding contrasts with previous findings that polypharmacy is linked to lower adherence (15, 16). In our study, non-adherent patients with bipolar disorder take more tranquilizers than adherent patients. This finding should make us more vigilant in prescribing tranquilizers, especially to patients who have the risk factors for less adherent behavior. Surprisingly, in our study adverse effects did not seem to have a major impact on medication adherence. In contrast, other studies found an association between adverse effects and non-adherence in patients with schizophrenia, bipolar disorder, and unipolar depression (14, 18, 47–50). Our study mostly comprises patients with chronic disorders and a longer history of medication intake. Adverse effects often occur when new medication is introduced or established medication is altered. This might explain why we could not find an association between adverse effects and non-adherence in our study.

Patients with a higher score for environmental quality of life also adhered better to their medication. This domain includes financial resources; a feeling of freedom; physical safety and security; accessibility and quality of health and social care; home environment; opportunities for acquiring new information and skills; participation in and opportunities for recreation; and physical environment, such as pollution, noise, traffic, climate, and transport (41). In the field of somatic diseases, adherence behavior was shown to increase the quality of life of patients with hypertension (51). Nevertheless, we have to be careful when interpreting findings such as this one because the cause and effect relationship may be unclear.

Additionally to our logistic regression analysis, we observed the following results in our explorative univariate analysis: Patients with male sex and younger age tended to be less adherent. Other studies also found that these characteristics are risk factors for poor adherence to treatment (3, 4, 52). In the univariate analysis, there was an association between higher crystallized intelligence, higher educational level, and adherent behavior. However, we did not investigate the relationship between cognitive deficits and adherence behavior in detail. Many studies point to an association between cognitive deficits and reduced medication adherence, but little is known about the educational level of non-adherent patients (53).

Despite the interesting findings and strengths of our study, some limitations should be discussed. The imputation of missing values might have added a small amount of additional uncertainty, and having a complete data set might also have improved the reliability of the results. No adjustment for multiple testing was implemented, given the exploratory nature of the analysis (54). Therefore, significant results have only exploratory character and a confirmatory validation of these results is necessary in further studies. In contrast to previous research results (4), only one fifth of the patients showed non-adherent behavior in our sample. Our sample consisted mainly of chronically ill patients. Patients with first episodes of a psychiatric disorder were underrepresented. These patients are typically younger and are considered to be less adherent (3, 4, 52). Patients who were asked to be included in the PsyCourse study are originally asked to participate in a longitudinal study. This might lead to a selection bias since the participation in a longitudinal study requires a high level of conscientiousness itself. Another limitation is that we evaluated adherence and the other factors both at the time of study enrolment. Therefore, the variables might have interacted with each other and cannot be interpreted independently. We only investigated medication adherence with a non-standardized, self-report questionnaire that focused on the regularity of medication intake and did not evaluate other important information, such as insight into the illness, attitude toward medication, and the perceived effect of the medication. Psychiatric treatment consists of much more than pure medication intake, for example other treatment forms, such as psycho- and sociotherapy; however, we were unable to investigate this aspect because the respective data were not available. Therefore, prospective studies and randomized controlled trials are needed to investigate the full picture of psychiatric care and treatment and respective patient adherence.

Taking our explorative results into consideration might help to identify those patients who are more adherent and those who require extra care in the form of psychoeducation, shared-decision making, and more frequent psychiatric consultations. Such an approach might help health care providers to determine personalized therapeutic strategies that facilitate adherence in patients at higher risk of discontinuing their medication.

## Data Availability Statement

The raw data supporting the conclusions of this article will be made available by the authors, without undue reservation.

## Ethics Statement

The studies involving human participants were reviewed and approved by Ethikkommission bei der Medizinischen Fakultät der LMU München. The patients/participants provided their written informed consent to participate in this study.

## Author Contributions

S-KK and FS designed, wrote, and revised the manuscript. ML contributed the statistics (multivariable analysis). ECS, JK, MB, and UH revised the analysis and its interpretation in detail. All authors contributed to the data collection of the PsyCourse study and revised and approved the final version.

## Funding

TS was supported by the Deutsche Forschungsgemeinschaft (DFG) within the framework of the projects www.kfo241.de and www.PsyCourse.de (SCHU 1603/4-1, 5-1, 7-1, FA241/16-1) and by the German Federal Ministry of Education and Research (BMBF) through the Integrated Network IntegraMent (Integrated Understanding of Causes and Mechanisms in Mental Disorders), under the auspices of the e:Med Programme (01ZX1614K). TS received additional support from the BMBF within the framework of the BipoLife network (01EE1404H) and the Dr. Lisa Oehler Foundation, Kassel (Germany).

## Conflict of Interest

AH has been invited to scientific meetings by Lundbeck, Janssen, and Pfizer, and he received paid speakerships from Janssen, Otsuka, and Lundbeck. He was member of Roche, Otsuka, Lundbeck, and Janssen advisory boards. AS was an honorary speaker for TAD Pharma and Roche and a member of Roche advisory boards. PF has been an honorary speaker for AstraZeneca, Bristol Myers Squibb, Lilly, Essex, GE Healthcare, GlaxoSmithKline, Janssen Cilag, Lundbeck, Otsuka, Pfizer, Servier, and Takeda and has been a member of the advisory boards of Janssen-Cilag, AstraZeneca, Lilly, Lundbeck, Richter, Recordati and Boehringer Ingelheim. CK received fees for an educational program from Aristo Pharma, Janssen-Cilag, Lilly, MagVenture, Servier, and Trommsdorff as well as travel support and speakers honoraria from Aristo Pharma, Janssen-Cilag, Lundbeck, Neuraxpharm and Servier. JZ was employed by Psychiatrieverbund Oldenburger Land gGmbH. The remaining authors declare that the research was conducted in the absence of any commercial or financial relationships that could be construed as a potential conflict of interest.

## Publisher's Note

All claims expressed in this article are solely those of the authors and do not necessarily represent those of their affiliated organizations, or those of the publisher, the editors and the reviewers. Any product that may be evaluated in this article, or claim that may be made by its manufacturer, is not guaranteed or endorsed by the publisher.
