# Supplementary material for: Medication Adherence in a Cross-Diagnostic Sample of Patients From the Affective-to-Psychotic Spectrum: Results From the PsyCourse Study
Source: Front Psychiatry. 2022 Jan 20;12:713060. doi: 10.3389/fpsyt.2021.713060 (PMC8811370; doi:10.3389/fpsyt.2021.713060)
Supplement: Supplementary file 1 [file Table_1.DOCX]

Supplementary Material

**Supplementary Table 1.** Variables and number of missing variables included in the multivariate analysis.

| **Variable** | **% missing** |
| --- | --- |
| Number of cigarettes | 2.3 |
| Sex | 0 |
| Current partner | 2.3 |
| Big Five: Extraversion | 8.8 |
| Big Five: Neuroticism | 8.8 |
| Big Five: Openness | 9.4 |
| Big Five: Conscientiousness | 9.3 |
| Big Five: Agreeableness | 8.9 |
| Crystallized IQ: Multiple-Choice Vocabulary Intelligence Test | 16.7 |
| Illness severity | 1.2 |
| Global Assessment of Functioning | 1.3 |
| Alcohol in past 12 months | 4.9 |
| 5 or more alcoholic beverages in past 12 months | 5.5 |
| Lifetime alcohol dependence | 9.8 |
| Illicit drugs | 4.1 |
| Antidepressants | 0 |
| Antipsychotics | 0 |
| Mood stabilizers | 0 |
| Tranquilizers | 0 |
| Other psychiatric medication | 0 |
| Adverse events under current medication | 29.3 |
| Ever had suicidal ideation | 2.6 |
| Suicide attempt | 2.8 |
| Number of suicide attempts | 4.7 |
| First-episode patient | 4.8 |
| Ever treated as inpatient or day patient for any mental health reason | 0.2 |
| Ever treated as outpatient for any mental health reason | 0.9 |
| Current psychiatric treatment | 1.0 |
| Country of birth | 0.8 |
| Currently impaired to exercise profession because of psychological/psychiatric symptoms | 6.5 |
| Disability pension because of psychological/psychiatric illness | 25.9 |
| Currently in paid employment | 0.9 |
| Professional education | 1.1 |
| Number of half siblings | 13.7 |
| Number of siblings | 6.3 |
| Number of biological children | 0.7 |
| Age | 0 |
| Age at first inpatient treatment for any mental health reason | 4.4 |
| Age at first outpatient treatment for any mental health reason | 7.3 |
| Diagnostic group | 0.6 |
| WHOQOL-BREF domain scores: Global | 8.5 |
| WHOQOL-BREF domain scores: Physical health | 9.9 |
| WHOQOL-BREF domain scores: Psychological | 10.3 |
| WHOQOL-BREF domain scores: Social relationships | 8.7 |
| WHOQOL-BREF domain scores: Environment | 9.9 |
